# Awareness, Interest, and Preferences of Primary Care Providers in Using Point-of-Care Cancer Screening Technology

**DOI:** 10.1371/journal.pone.0145215

**Published:** 2016-01-15

**Authors:** Chloe S. Kim, Sarah Vanture, Margaret Cho, Catherine M. Klapperich, Catharine Wang, Franklin W. Huang

**Affiliations:** 1 Department of Biomedical Engineering, Boston University, Boston, Massachusetts, United States of America; 2 Department of Community Health Sciences, School of Public Health, Boston University, Boston, Massachusetts, United States of America; 3 Harvard Medical School, Boston, Massachusetts, United States of America; 4 Dana-Farber Cancer Institute, Boston, Massachusetts, United States of America; University of South Australia, AUSTRALIA

## Abstract

Well-developed point-of-care (POC) cancer screening tools have the potential to provide better cancer care to patients in both developed and developing countries. However, new medical technology will not be adopted by medical providers unless it addresses a population’s existing needs and end-users’ preferences. The goals of our study were to assess primary care providers’ level of awareness, interest, and preferences in using POC cancer screening technology in their practice and to provide guidelines to biomedical engineers for future POC technology development. A total of 350 primary care providers completed a one-time self-administered online survey, which took approximately 10 minutes to complete. A $50 Amazon gift card was given as an honorarium for the first 100 respondents to encourage participation. The description of POC cancer screening technology was provided in the beginning of the survey to ensure all participants had a basic understanding of what constitutes POC technology. More than half of the participants (57%) stated that they heard of the term “POC technology” for the first time when they took the survey. However, almost all of the participants (97%) stated they were either “very interested” (68%) or “somewhat interested” (29%) in using POC cancer screening technology in their practice. Demographic characteristics such as the length of being in the practice of medicine, the percentage of patients on Medicaid, and the average number of patients per day were not shown to be associated with the level of interest in using POC. These data show that there is a great interest in POC cancer screening technology utilization among this population of primary care providers and vast room for future investigations to further understand the interest and preferences in using POC cancer technology in practice. Ensuring that the benefits of new technology outweigh the costs will maximize the likelihood it will be used by medical providers and patients.

## Introduction

Point-of-care (POC) testing is defined as “testing at or near the site of patient care whenever the medical care is needed”. POC testing can play an important role in cancer care, which is a continuous process from prevention, screening, diagnosis, treatment to survivorship. Appropriate and timely treatment decisions can be made when physicians are given immediate information about patients’ condition by using POC testing, which will lead to reduced patients’ morbidity, mortality, criticality, and therefore increase the chances of survival [1,2]. As the cancer care continuum includes multiple technical stages, communication steps, and interactions between patients, providers, and organizations, using POC testing can help reduce the number of steps in each process and help ensure more patients stay within the system to receive the care they need [3,4].

POC testing has the potential to resolve issues such as inconvenient scheduling times, long waits, and variability in healthcare services that are reported barriers in accessing follow-up care for cancer [2]. The goal of POC testing is to produce rapid results without the need for repeated visits to facilitate the timely implementation of appropriate treatment [5,6]. POC technology may be able to reduce the number of steps in the care continuum by bringing the test closer to the patient care site, and by removing reported barriers, as well as shortening the turnaround time and facilitating clinical management decisions [3,7].

The existing examples of POC technology demonstrate its potential for better disease monitoring and control for infectious disease, diabetes, and cancer in low- and high-resource settings [8]. Various novel POC cancer screening tools are now available for use in practices around the world. For example, Digital Breast Tomosynthesis, which is a new way of screening for breast cancer using 3-D mammograms, is being rapidly implemented in breast imaging clinics around the world [9]. A pilot-study to measure sensitivity of a mobile POC system for measuring breast cancer biomarkers for breast cancer screening has been shown to predict women with breast cancer and abnormal mammograms, which suggests the potential to reduce unnecessary mammograms without losing diagnostic sensitivity [10].

Located at Boston University School and funded by National Institute of Biomedical Imaging and Bioengineering, the Center for Future Technology in Cancer Care (CFTCC) is seeking to improve the quality of cancer care through the identification and prototyping of innovative point of care technologies. Conducting user research and the integration of the results into product development are well-recognized important factors in medical device development [11]. In order to ensure that newly developed cancer screening POC technologies meet existing needs and expectations of medical providers, CFTCC surveyed primary care providers including primary care physicians, family physicians, and advanced practice nurses to assess their level of awareness, interest, and preferences in using POC cancer screening technology in their practice and to provide guidelines to biomedical engineers for future POC technology development.

## Methods

### Study Population/Recruitment

The survey was distributed through the following three outlets– 1) the link to the online survey was forwarded to primary care providers by Boston Medical Center and two of its affiliated community health centers, and also by the Department of Public Health LA County, 2) the link to the survey was forwarded to the members in the Center for Integration of Medicine and Innovative Technology (CIMIT) listserv, 3) it was posted on the CFTCC website (http://www.bu.edu/cftcc/category/cftcc-survey/). The recruitment letter stated that the survey was only for primary care providers including primary care physicians, family physicians, and nurse practitioners. To encourage people to participate, a $50 Amazon Gift Card was provided as an honorarium for the first 100 respondents. This study was exempted by Institutional Review Board (IRB) of Boston University. The survey was distributed throughout the second and third week of January 2014 and deactivated on February 24, 2014.

### Survey Instrument

We developed a 27-item survey, which used a Likert response, multiple choice and write-in responses. Participants were asked to rate their agreement with statements such as “it is my first time hearing the term point-of-care technology”, “I know what point-of-care cancer screening technology is”, and “I understand why point-of-care cancer screening technology could be useful”. Two questions were asked to rank potential advantages and important features of POC cancer screening technology, and two questions to rank provider- and system-level barriers to providing cancer prevention care.

The level of interest in using POC cancer screening technology was assessed by asking the participants to choose one of the four options–“not interested at all”, “not very interested”, “somewhat interested”, and “very interested”. The outcome of interest was dichotomized with “very interested” as one group, and the “somewhat interested”, “not very interested” and “not interested at all” as one group.

Based on the general description of POC technology, we defined “POC cancer screening technology” as “a cancer screening test occurring at the point where patient care is given, wherever that is located; for example, patient’s bedside, pharmacy, physician’s office, or patient’s home, instead of having to be referred to a different location and/or at a different time” [1,12]. The definition of POC was provided at the very beginning of the survey to ensure all participants had a basic understanding of what constitutes POC technology.

### Data Analysis

Data were described with frequencies and percentages in order to measure clinicians’ awareness, interest, and preferences in using POC. Chi-square analyses were used to test associations between various independent variables and the outcome variable “interest of using POC cancer screening technology” and “understanding why POC could be useful”. Multivariate logistic regression analysis was used to analyze the effect of multiple demographic variables on various outcomes of interest. Odds ratios, 95% confidence intervals, and p-values were reported for each association, and p-values less than 0.05 were considered to be significant. Data were analyzed using SAS 9.3.

## Results

### Respondent Characteristics

Overall, 415 subjects began the online survey. Sixty-five participants (16%) were excluded from the analysis because they either did not complete the survey and/or were not a primary care provider. Three hundred fifty people were included in the final analyses. Demographic characteristics of 350 participants are presented in Table 1. Over 40% of the respondents were from a non-academic hospital, while 33% and 25% of the respondents were from an academic hospital and community health center, respectively. Most of the participants (97%) reported to have at least 25% of their patients on Medicaid. Seventy five percent of participants stated that they spent more than 5 minutes discussing cancer screening recommendations with their patients.

**Table 1:** Demographic Characteristics of Study Participants.

Characteristics	Primary Care Physician (%) (n = 269)	Advanced Nurse Practitioner (%) (n = 81)	All Respondents (%) (N = 350)
Gender			
Male	170 (63.20)	26 (32.10)	196 (56.00)
Female	99 (36.80)	55 (67.90)	154 (44.00)
Organization			
Academic Hospital	98 (36.43)	19 (23.46)	117 (33.43)
Non-Academic	110 (40.89)	32 (39.51)	142 (40.57)
CHC	60 (22.30)	29 (35.80)	89 (25.43)
Other	1 (0.37)	1 (1.23)	2 (0.57)
Years in Practice			
0–5 yrs	47 (17.47)	10 (12.35)	57 (16.29)
6–15 yrs	108 (40.15)	35 (43.21)	143 (40.86)
16–25 yrs	83 (30.86)	27 (33.33)	110 (31.43)
26–35 yrs	29 (10.78)	7 (8.64)	36 (10.29)
Over 36 yrs	2 (0.74)	2 (2.47)	4 (1.14)
Patients on Medicaid			
Less than 25%	8 (2.99)	1 (1.23)	9 (2.58)
25%-50%	79 (29.48)	25 (30.86)	104 (29.80)
51%-75%	141 (52.61)	42 (51.85)	183 (52.44)
More than 75%	39 (14.55)	12 (14.81)	51 (14.61)
I don’t know	1 (0.37)	1 (1.23)	2 (0.57)
Number of Patients per Day			
0–10 patients	15 (5.58)	8 (9.88)	23 (6.57)
11–20 patients	94 (34.94)	21 (25.93)	115 (32.86)
21–30 patients	106 (39.41)	35 (43.21)	141 (40.29)
31–40 patients	36 (13.38)	13 (16.05)	49 (14.00)
More than 40	18 (6.69)	4 (4.94)	22 (6.29)
Time Spent per Patient			
Less than 10 mins	3 (1.12)	2 (2.47)	5 (1.43)
11–20 mins	105 (39.03)	26 (32.10)	131 (37.43)
21–30 mins	118 (43.87)	40 (49.38)	158 (45.14)
More than 30 mins	43 (15.99)	13 (16.05)	56 (16.00)
Time Spent for Cancer Discussion			
0 min	2 (0.74)	0 (0)	2 (0.57)
1–5 mins	79 (29.37)	9 (11.11)	88 (25.14)
5–10 mins	145 (53.90)	46 (56.79)	191 (54.57)
More than 10 mins	43 (15.99)	26 (32.10)	69 (19.71)

### Awareness and Interest in Using POC Cancer Screening Technology

Three survey questions were asked to assess primary care providers’ awareness of POC cancer screening technology. More than half of the participants (57%) stated that they heard of the term “POC technology” for the first time when they took the survey (Table 2). However, after reading the provided definition of POC cancer screening technology, 89% of the respondents reported to understand the purpose and usefulness of POC technology. Participants were asked to choose their interest level of using POC cancer screening technology in their practice. Over two-thirds of participants (68%) expressed that they were “very interested” in using POC cancer screening tests in their practice, while 29% said they were “somewhat interested”, and only 2% stated they were “not very interested”. Among those participants who expressed their interest in using POC screening test, 43% of them chose “no need to refer” and another 43% chose “better cancer prevention care will be provided” as their primary reasons for being interested in POC technology.

**Table 2:** Knowledge of and Interest in Using POC Technology.

Variables	All Respondents
This is my first time hearing POC	
Strongly Agree	113 (32.29)
Agree	86 (24.57)
Disagree	67 (19.14)
Strongly Disagree	84 (24.00)
I know what POC is	
Strongly Agree	168 (48.00)
Agree	145 (41.43)
Disagree	29 (8.29)
Strongly Disagree	8 (2.29)
I understand why POC could be useful	
Strongly Agree	186 (53.30)
Agree	126 (36.10)
Disagree	33 (9.46)
Strongly Disagree	4 (1.15)
How interested would you be in using a new point-of-care cancer screening technology in your practice if it were available?	
Very Interested	234 (68.22)
Somewhat Interested	100 (29.15)
Not Very Interested	7 (2.04)
Not Interested At All	2 (0.58)
Primary reason for being interested in using a cancer screening point-of-care technology	
No need to refer	145 (43.41)
Not satisfied with current methods	44 (13.17)
Better cancer prevention care will be provided	145 (43.41)
Ideal way to communicate the test results when result is positive	
In person	145 (42.27)
On the phone	75 (21.87)
Via email	93 (27.11)
Interactive smartphone app	30 (8.75)
Ideal way to communicate the test results when result is negative	
In person	133 (38.78)
On the phone	84 (24.49)
Via email	85 (24.78)
Interactive smartphone app	41 (11.95)

### Interest in Using Interactive Smartphone Applications for Communicating Test Results

For both positive and negative cancer screening test results, respondents preferred in-person communication (42% and 39%, respectively) over phone, email, or smartphone application (Table 2). Using an “interactive smartphone app” to communicate positive and negative screening test results was least preferred by study participants (9% and 12%, respectively).

### Needs and Barriers to Providing Cancer Care

Having to discuss competing health risks with their patients (35%) was shown to be the most significant provider-level barrier in providing cancer care, followed by lack of support staff (21%) and lack of result feedback (18%) (Table 3). Difficulty scheduling for a screening test (32%) was the most significant system-level barrier followed by multiple steps/days for screening test (24%) and lack of efficient follow-up/monitoring system (23%). Colorectal cancer (41%) was chosen to be the cancer type that clinicians reported having the greatest need for improvement with the help of POC technology followed by breast cancer (34%) and prostate cancer (24%). The most significant advantages of new POC technology included rapid data availability (31%), user-friendly (29%), and potential cost-savings (24%), while the most important features included finding cancer in its early stage (37%) and distinguishing aggressive from benign cancer (30%).

**Table 3:** Needs and Barriers to Providing Cancer Care.

Measure	All Respondents
Most important potential advantage of POC technology	
Rapid data availability	108 (31.49)
User-friendly	99 (28.86)
Potential cost-savings	81 (23.62)
Making screening a one-time visit	33 (9.62)
Removing intermediary	22 (6.41)
Most important feature of POC technology	
Finding cancer in its early stage	124 (36.58)
Distinguishing aggressive vs. benign cancer	101 (29.79)
Requiring a short amount of time for screening	56 (16.52)
Not interrupting current workflow	39 (11.50)
Easy interpretation of the results	19 (5.60)
Most significant provider-level barrier	
Having to discuss competing health risks	118 (34.71)
Lack of support staff	72 (21.18)
Lack of screening reminders	52 (15.29)
Lack of result feedback	61 (17.94)
Lack of knowledge on current recommendations	37 (10.88)
Most significant system-level barrier	
Difficulty scheduling for screening test	111 (32.46)
Multiple steps/days for screening test	83 (24.27)
Lack of efficient follow-up/monitoring system	79 (23.10)
Lack of social protection for medical leave for patients	42 (12.28)
Lack of cancer prevention education material	27 (7.89)
Which cancer type has most room for improvement	
Breast	117 (34.41)
Colorectal	140 (41.18)
Prostate	83 (24.41)

### Factors Impacting the Level of Interest in Using POC technology

A bivariate analysis showed respondents who were exposed to the term POC for the first time when taking the survey had 1.64 times the odds [95% CI (1.04–2.60), p-value = 0.04] of being “very interested” in using POC cancer screening technology when compared to those who had previously heard of the term. Participants with basic understanding of POC had 1.96 times the odds [95% CI (0.96–3.97), p-value = 0.06] of being strongly interested in using POC compared to those participants without basic understanding. In addition, participants who said they understood the usefulness of POC had 5.25 times the odds [95% CI (2.51–10.96), p-value <0.0001] of being strongly interested in using POC compared to those who said they did not know its usefulness (Table 4). However, a multivariate analysis including all significant bivariate findings found only “understanding” to still be a significant predictor of interest with those who understood why POC could be useful having 5.05 times the odds [95% CI (2.36–10.83), p-value<0.0001] of being “very interested” in using POC cancer screening technology (Table 5).

**Table 4:** Bivariate Analysis of Interest Level in Using POC.

Variables	N (Percent)	OR	95% CI	P-Value
First time hearing the term POC				
Yes	199 (58.85)	1.6409	1.0375–2.5953	0.0355
No	145 (41.15)			
I know what POC is				
Yes	309 (89.83)	1.9559	0.9634–3.9705	0.0598
No	35 (10.17)			
I understand why POC could be useful				
Yes	308 (89.53)	5.2471	2.5119–10.9603	<.0001
No	36 (10.47)			
How long have you been in the practice of medicine?				
0–5 years (reference)	56 (16.28)	1	-	-
6–15 years	142 (41.28)	1.004	0.512–1.967	0.9917
16–25 years	107 (31.10)	0.825	0.412–1.653	0.5867
26 years or more	39 (11.34)	0.981	0.404–2.381	0.9658
What percentage of your patient population is on Medicaid?				
< = 50% (reference)	109 (31.78)	1	-	-
51%-75%	183 (53.35)	0.843	0.508–1.399	0.5084
> = 75%	51 (14.87)	1.648	0.755–3.599	0.2095
How many patients do you see per day?				
0–10 patients	22 (6.40)	1	-	-
11–20 patients	110 (31.98)	0.656	0.238–1.812	0.4162
21–30 patients	141 (40.99)	0.827	0.303–2.255	0.7102
31–40 patients	49 (14.24)	1.462	0.4553–4.700	0.5233
More than 40 patients	22 (6.40)	0.542	0.153–1.921	0.3425
How much time do you spend with a patient per visit during an annual check-up?				
< = 20 minutes (reference)	134 (38.95)	1	-	-
21–30 minutes	156 (45.35)	1.059	0.642–1.746	0.823
> = 30 minutes	54 (15.70)	0.776	0.400–1.505	0.4529
How much time do you or members of your team spend discussing cancer screening recommendations?				
< = 5 minutes (reference)	86 (25.00)	1	-	-
5–10 minutes	189 (54.94)	1.808	1.058–3.090	0.0302
> = 10 minutes	69 (20.06)	1.466	0.754–2.849	0.2589
Gender				
Male	192 (55.81)	0.6901	0.4372–1.0894	0.1294
Female	152 (44.19)			
Position				
Primary care physician	264 (76.74)	1.108	0.6435–1.9079	0.7844
Advanced nurse practitioner	80 (23.26)			
Organization type				
Academic medical center (reference)	115 (33.43)	1	-	-
Hospital–non-academic	142 (41.28)	0.99	0.585–1.675	0.97
Community health center	87 (25.29)	1.113	0.609–2.034	0.7281

**Table 5:** Multivariate Analysis of Interest Level in Using POC.

Variables	N (Percent)	OR	95% CI	P-Value
First time hearing the term POC				
Yes	199 (58.85)	1.513	0.928–2.468	0.0969
No	145 (41.15)			
I understand why POC could be useful				
Yes	308 (89.53)	5.051	2.357–10.825	<.0001
No	36 (10.47)			
How much time do you or members of your team spend discussing cancer screening recommendations?				
< = 5 minutes (reference)	86 (25.00)	1	-	-
5–10 minutes	189 (54.94)	1.698	0.966–2.984	0.0316
> = 10 minutes	69 (20.06)	0.999	0.496–2.012	0.3869

The length of practicing medicine, the percentage of Medicaid patients, the average number of patients per day, and the time spent with a patient per annual visit were not shown to be associated with the level of interest in using POC. In the bivariate analysis, clinicians who spend between five to ten minutes discussing cancer-related issues with their patients were more likely to be “very interested” in using POC in their practice compared to those who spend less than or equal to five minutes [p-value = 0.03]. In the multivariate model, the 5–10 minute group appeared to be a significant predictor of interest [p-value = 0.03], however this group was not statistically different in interest level [OR = 1.70, 95% CI (0.97–2.98)] when compared to those who spend less than 5 minutes or more than 10 minutes discussing cancer screening recommendations (Table 5). Gender, current position, and organization type were not shown to be associated with the level of clinicians’ interest in using POC.

### Factors Impacting Understanding Why POC Could be Useful

A bivariate analysis of factors impacting understanding of why POC could be useful showed respondents who expressed they knew what POC was had 35.66 times the odds [95% CI (14.93–85.15), p-value<0.0001] of understanding why POC could be useful. Additionally, those who were exposed to the term POC for the first time when taking the survey had 2.70 times the odds [95% CI (1.32–5.53), p-value = 0.007] of understanding why POC could be useful when compared to those who had heard the term before. Advanced nurse practitioners had 0.33 times the odds [95% CI (0.16–0.67), p-value = 0.002] of understanding why POC could be useful when compared to primary care physicians (Table 6). However, in the multivariate logistic regression analysis of these variables only position appeared to be a significant predictor of understanding why POC could be useful. Advanced Nurse Practitioners had 0.34 times the odds [95% CI (0.15–0.71), p-value = 0.005] of understanding why POC could be useful when compared to Primary Care Physicians indicating physicians were more likely to understand why POC could be useful (Table 7).

**Table 6:** Bivariate Analysis of understanding why POC could be useful.

Variables	N (Percent)	OR	95% CI	P-Value
I know what POC is				
Yes	309 (89.83)	35.6593	14.9342–85.1460	<.0001
No	35 (10.17)			
First time hearing the term POC				
Yes	308 (89.53)	2.6974	1.3164–5.5271	0.0070
No	36 (10.47)			
How long have you been in the practice of medicine?				
0–5 years (reference)	56 (16.28)	1	-	-
6–15 years	142 (41.28)	0.339	0.074–1.541	0.6539
16 years or more	146 (42.44)	0.233	0.053–1.033	0.0448
What percentage of your patient population is on Medicaid?				
< = 50% (reference)	109 (31.78)	1	-	-
51%-75%	183 (53.35)	0.926	0.411–2.086	0.4992
> = 75%	51 (14.87)	0.53	0.196–1.438	0.1736
How many patients do you see per day?				
0–10 patients	22 (6.40)	1	-	-
11–20 patients	110 (31.98)	0.686	0.144–3.257	0.0852
21–30 patients	141 (40.99)	0.683	0.147–3.173	0.0731
31–40 patients	49 (14.24)	4.798	0.411–55.951	0.139
More than 40 patients	22 (6.40)	2.1	0.176–25.010	0.6157
How much time do you spend with a patient per visit during an annual check-up?				
< = 20 minutes (reference)	134 (38.95)	1	-	-
21–30 minutes	156 (45.35)	0.841	0.404–1.750	0.2089
> = 30 minutes	54 (15.70)	1.983	0.546–7.199	0.2156
How much time do you or members of your team spend discussing cancer screening recommendations?				
< = 5 minutes (reference)	86 (25.00)	1	-	-
5–10 minutes	189 (54.94)	1.69	0.824–3.466	0.9603
> = 10 minutes	69 (20.06)	3.018	-	-
Gender				
Male	192 (55.81)	0.9884	0.4934–1.9800	0.9737
Female	152 (44.19)			
Position				
Advanced nurse practitioner	80 (23.26)	0.3279	0.1608–0.6687	0.0015
Primary Care Physicians	264 (76.74)			
Organization type				
Academic medical center (reference)	115 (33.43)	1	-	-
Hospital–non-academic	142 (41.28)	0.336	0.130–0.867	0.0667
Community health center	87 (25.29)	0.424	0.148–1.215	0.4502

**Table 7:** Multivariate Analysis of understanding why POC could be useful.

Variables	N (Percent)	OR	95% CI	P-Value
I know what POC is				
Yes	309 (89.83)	30.471	12.525–74.130	<.0001
No	35 (10.17)	-	-	-
First time hearing the term POC				
Yes	308 (89.53)	2.418	1.153–5.071	0.0195
No	36 (10.47)	-	-	-
Position				
Advanced nurse practitioner	80 (23.26)	0.322	0.147–0.705	0.0046
Primary Care Physicians	264 (76.74)	-	-	-
How long have you been in the practice of medicine?				
0–5 years (reference)	56 (16.28)	1	-	-
6–15 years	142 (41.28)	0.365	0.079–1.677	0.5159
16 years or more	107 (31.10)	0.246	0.055–1.101	0.0509

Percentage of patient population on Medicaid, number of patients seen per day, time spent with a patient during annual check-up, time spent discussing cancer screening recommendations, gender, and organization type were not shown to be associated with understanding why POC could be useful (Table 6). In the bivariate model, length of time spent practicing medicine was shown to be a significant predictor of understanding why POC could be useful for those practicing 16 years or more [p-value = 0.04]. However, this group did not demonstrate a higher likelihood of understanding why POC could be useful when compared to those who practiced medicine for less than 16 years [OR = 0.23, 95% CI (0.05–1.03)] and was not significant in the multivariate analysis (Tables 6 and 7).

## Discussion

The potential benefits of POC cancer screening technology are clear both nationally and globally [8,13]. As the result of a rapidly growing interest of POC technology, biomedical engineers may become easily attracted to developing new technologies before carefully considering preferences and needs of clinicians. Our study findings may help medical communities, as well as POC cancer screening technology developers, to have better understandings of how POC cancer testing is perceived by medical providers before committing their resources on adopting or developing such technologies.

There seems to be a lack of exposure to the concept of POC technology among our participants as a majority (57%) of participants heard the term POC cancer screening technology for the first time when they took this survey. However, the vast majority of the participants (97%) expressed interest in using POC technology after being given the description of POC and how it could potentially be useful in their practice. This data suggests the potential for well-developed POC cancer screening technology to be accepted and adopted in primary care settings if designed to overcome some of the existing barriers to providing cancer care including cost, legality, time, fear, usefulness, and complexity [5].

The lack of clinicians’ interest in using mobile applications in communicating with their patients was shown in our result. Some mobile phone-based applications have shown to improve health outcomes for various health conditions, which sparked engineers to pursue the development of new mobile medical applications [14]. Mobile applications can be used for various purposes by medical providers such as the direct provision of care, real-time monitoring of patient vital signs, delivery of patient information to practitioners, and collection of data [15]. Using mobile applications can also help patients become more committed to the healthy and cancer-preventive lifestyle, which is a very significant factor in cancer development [16]. With the development of advanced cell-phone technologies, tailoring information in real time according to individuals’ needs has become possible [17]. However, our results suggest that primary care providers prefer in-person communication over technology-dependent communication methods for conveying positive and negative test results. This preference is easily understandable for communicating positive results as physicians will need to further explain the test results and next steps with the patients. It will be interesting for researchers to investigate why mobile applications are not preferred by medical providers even for communicating negative test results and what new approaches must be taken to create mobile applications that meet the needs of medical providers.

Our study results also revealed the importance of defining the scope of POC cancer screening technology in setting the appropriate expectations of end-users of the technology. System-level barriers, such as difficulty scheduling for a screening test, multiple steps/days to obtain a screening result, and the lack of efficient follow-up/monitoring system, could be addressed with POC technology by providing cancer care at the point where patient care is given. By performing the screening test where patient care is given, patients no longer need to wait for the specialist to become available, or return to the hospital for the screening test, and clinicians do not need to follow-up with patients to monitor their screening status. However, some of the provider-level barriers recognized by our study participants, such as having to discuss competing health risks with patients and lack of support staff, fall outside of the current scope of POC technology. Systemic changes must be made in order to resolve these provider-level barriers.

Through our study, we wanted to deliver insights and guidelines to clinicians and biomedical engineers as they continue to adopt and develop new POC cancer screening technology. According to our survey results, medical providers believe that there is a need for a cancer screening test with rapid data availability and ability to find cancer in its early stage. These preferences of medical providers must be considered in developing a new POC technology in order to allow the new technology to be widely adopted in current medical practice. Similar survey studies should be performed for specific prototypes in order to gain more detailed information for such devices to be welcomed by the medical community.

An interesting study finding was that clinicians were most interested in developing POC technology for colorectal cancer compared to breast and prostate cancer. Among the three cancer types included in this survey, prostate cancer screening test has been most controversial, which resulted in the continuing search for a better biomarker than PSA [18]. Therefore, we hypothesized that there would be more interest in developing a new POC technology for prostate cancer than for breast or colorectal cancer. However, our results show that primary care providers consider colorectal cancer as an area of need for POC technology. One possibility for this finding is that even though a colonoscopy is widely accepted as an effective screening tool, there are other factors such as patients not following up after referral to obtain a colonoscopy, their inability to afford one, or fear of pain that clinicians believe that POC technology may be able to address for colorectal cancer [19,20].

We found that primary care providers who understood why POC technology could be useful were more likely to be interested in using POC technology compared to providers who did not. The possible explanation for this association is that providers who value POC technology by believing it to be useful are likely to have their value of POC technology extend to interest in its further use. A previous study completed on physician and medical technology demonstrated “perceived usefulness” as a strong predictor of physician interest in use of medical technology (23). Our study results reinforce this finding with understanding why POC technology could be useful being the strongest statistical predictor of interest in POC. Potential end-users of POC cancer screening technology must be given a thorough explanation of benefits and drawbacks of the technology for them to make an informed decision about using such technology in their practice.

There are several limitations to our study. The nature of the online survey form makes it difficult to estimate how many people were invited to participate in the survey and to verify the demographic information of the participants. Additionally, this distribution method limits our ability to estimate response rates among the participating hospitals and community centers. Those who answered may have been more interested in POC technologies, thus contributing to the high level of interest observed in the survey. Furthermore, we did not collect information regarding respondent’s prior use of POC technology, a variable that may have provided an additional explanation for interest in POC technology.

Finally, in the definition of POC technology given at the beginning of the survey, no examples of POC technology were provided to respondents. While we believe this definition to be comprehensive, there is a risk that a respondent’s interpretation of our definition to actual devices in practice was not what we envisioned. No questions were asked to ascertain what specific devices respondents thought fell within our definition of POC technology, therefore we cannot be certain how they interpreted POC technology.

While there is a need to further investigate the benefits, drawbacks, and physicians’ preferences of POC cancer screening technology, as well as the existing clinical needs in cancer care, our results suggest that there is potential for POC cancer screening technology to be widely adopted in current medical practice. Future investigators should consider additional research to identify the cancer type-specific factors and desired attributes of a new POC technology that may influence medical providers’ decisions to utilize POC technology. More targeted questions related to a specific cancer type or a specific device should be asked to clinicians in order to provide more detailed, useful information to POC developers. Biomedical engineers could then utilize those data to ensure that development of a new POC technology addresses the existing clinical needs and preferences of medical providers in providing cancer care.

New medical devices and technologies are often welcomed by patients, patient’s family members, and medical providers because of their belief that new technologies, simply because they are new, may offer improved patient care. However, the development and introduction of new medical technology has shown to be one of the primary reasons behind the increased cost of health care delivery in the United States [21]. Advances in technology are only worth it when the benefits outweigh the costs [22]. Therefore, the cost of health care delivery also needs to be taken into account when developing a new POC technology in order for it to be widely utilized and bring benefits to the low-income, underserved populations.

In summary, there is a great interest in POC cancer screening technology utilization among primary care providers and growing opportunity for future investigations to take place to better understand the existing needs and interest in using POC cancer technology. Well-developed POC cancer screening tools will be able to provide better cancer care to patients in both developed and developing countries.
